# The Relationship between Cultural Anxiety and Ethnic Essentialism: The Mediating Role of an Endorsement of Multicultural Ideology

**DOI:** 10.1371/journal.pone.0141875

**Published:** 2015-11-10

**Authors:** Xiao-li Yang, Li Liu, Yuan-yuan Shi, Yong-shuai Li, Xuyun Tan, Xiao-meng Hu, Xiao-min Sun

**Affiliations:** 1 School of Psychology, Research Center for the Educational Development of Minorities, Northwest Normal University, Lanzhou, China; 2 School of Psychology, Beijing Normal University, Beijing, China; 3 Department of Psychology, Rutgers—The State University of New Jersey, New Brunswick, New Jersey, United States of America; Örebro University, SWEDEN

## Abstract

Many studies have explored the social consequences of ethnic essentialism in recent decades. In addition, a few studies have focused on the impact of perceived cultural context on ethnic essentialism. However, it is not clear why perceived cultural context can lead to changes in ethnic essentialism. In the present study, we hypothesized that the cultural anxiety of ethnic minorities may trigger a strong endorsement of and support for a multicultural ideology, thereby affecting beliefs about ethnic groups. To address the issue, 226 Tibetan and 102 Hui college students from Mainland China completed our questionnaires. The results across the two samples showed that (1) cultural anxiety was positively associated with both the endorsement of a multicultural ideology and ethnic essentialism, (2) cultural anxiety and the endorsement of a multicultural ideology positively predicted ethnic essentialism after controlling for demographic variables, and (3) cultural anxiety had both a direct effect on ethnic essentialism and an indirect effect on ethnic essentialism through the endorsement of a multicultural ideology. Our findings suggest that when ethnic minorities experience cultural anxiety, they might endorse a multicultural ideology and adopt essentialism to affirm their ethnic identities.

## Introduction

In recent decades, a growing number of studies have explored the causes and consequences of ethnic essentialism [1, 2]. Some studies have focused on the impact of the perceived cultural context on ethnic essentialism, showing that the perceived cultural context can raise ethnic essentialism [2]. Other studies have focused on the impact of ethnic essentialism on intergroup relations, showing that increased ethnic essentialism leads to group stereotyping [3, 4] and prejudice [5]. However, it is not clear why the perceived cultural context can lead to changes in ethnic essentialism. The present study seeks to address this issue. Inspired by research on cultural anxiety [6, 7, 8, 9] and an endorsement of a multicultural ideology [10, 11], we postulate that cultural anxiety is positively associated with ethnic essentialism and that the endorsement of a multicultural ideology mediates the relationship. The aim of the present study is to test these assumptions.

### Cultural Anxiety and Ethnic Essentialism

Cultural anxiety refers to individuals’ subjective sense of the risk that their ethnic culture could be changed and the resulting concern and worry about the development and survival of his/her ethnic cultural heritage [6, 8, 9]. Cultural anxiety is a critical issues faced by many people and countries in the process of social transition. Cultural anxiety, as a typical perceived cultural context, has become a general reality in modern society, especially for minority groups [7]. Ethnic Essentialism is an abbreviation of the psychological essentialism of an ethnic group, referring to people’s beliefs about their ethnic group sharing an inalterable essence [12, 13]. Past studies have shown that ethnic essentialism is negatively correlated with intergroup cognition, attitude and behavior. As beliefs about the essential nature of a group increase, attention to stereotype-consistent, as opposed to stereotype-inconsistent, information increases [14], along with endorsement of those stereotypes [3,5]. Ethnic essentialism is positively correlated with lower interest in intergroup contact and less concern for racial inequality in society [15], with more intergroup bias [16, 17], as well as less helping of other groups [15]. Studies have found that ethnic essentialism can amplify the perceived difference between different groups because stronger essentialist beliefs can lead to better performance in differentiating ethnic memberships [18].

There is a subtle link between cultural anxiety and ethnic essentialism. Terror Management Theory suggests that maternal cultural values are usually symbols of “immortal thinking”. Adhering to one’s own ethnic culture brings a feeling of having an "immortal soul", which can reduce people’s anxiety about threats to survival [19]. Research has shown that death anxiety is an expression of humans’ existential motivation [5], and this type of anxiety can cause group essentialism. Research has also shown that people’s development and maintenance of ethnic essentialism have underlying social motivations. Majority groups hold the beliefs of essentialism to rationalize their dominant status and social inequality [20]. In contrast, for minority groups, if their cultural identities are ignored/or discriminated against, they will exploit ethnic essentialism to affirm their cultural identities and fight for social equality [20]. It is shown that developing motivations and maintaining essentialist beliefs is an expression of existential motivations, e.g., buffering cultural anxiety [5]. Therefore, we infer that the stronger people’s cultural anxiety is, the more deeply they will believe that ethnic characteristics are natural and immutable. That is to say, the stronger the cultural anxiety is, the stronger the ethnic essentialism is (hypothesis 1).

### Endorsing a Multicultural Ideology as a Mediator

Endorsing a multicultural ideology refers to people’s belief in the existence of cultural diversity and the maintenance of different cultural identities within the same political framework [10, 21][]. Sidanius and Pratto[20] proposed the ideological asymmetry hypothesis, which implies that hierarchy-attenuating ideologies such as multiculturalism will appeal more to minority or low-status groups than to the majority or high status group. For minority groups, multiculturalism offers the possibility of maintaining their own culture and obtaining higher social status in society. Hence, minority group members should support multiculturalism more strongly than majority group members, especially when group interests are at stake and the minority group’s cultural value is threatened [20, 21]. The endorsement of multiculturalism is a collective strategy for dealing with a negative group identity [21] and for challenging group-based hierarchy and domination [21, 22]. Therefore, anxiety about their ethnic culture would lead ethnic minorities to further support and maintain a multicultural ideology. The stronger the attitude ethnic groups have toward endorsing a multicultural ideology, the more they will perceive their group sharing to be an inherent essence. The motive of maintaining a multicultural ideology can be understood as an important basis for the development and endorsement of essentialism. Essentialism clearly reflects people’s social motivations to maintain and confirm their ethnic cultural values [5]. In many countries, ethnic minority groups have put forward essentialist arguments for the legitimization of their ethnicity and culture [21]. We infer that the more anxiety groups have about their culture, the more they will endorse a multicultural ideology, which is accompanied by stronger ethnic essentialism. In other words, the endorsement of a multicultural ideology mediates the relationship between cultural anxiety and ethnic essentialism (hypothesis 2).

Tibetan and Hui people account for a large population of minorities in Northwest China. As of 2014 Census, the combined population of minority groups comprised 8.49% of the total population in China, including 0.49% of the Tibetan and 0.75 of the Hui. Tibetan is considered one of the most ancient nationalities in China. There are approximately 6.4 million Tibetans living in Chinese territory, most of which are distributed in a remote area of China, such as Tibet and Qinghai and Gansu Provinces. In addition, the entire population of the Tibetan people believes in Buddhism and uses their own characters and language. The Hui ethnic group is the most widely distributed minority group in mainland China, especially in Northwest China. Dating back to the 13^th^ century, the Mongol army conquered their territory westward, which resulted in a large number of people flooding into China to find refuge. As time went on, absorbing different ethnic compositions from Han, Mongolian, and Uyghur, this group of people has gradually evolved into a completely new ethnic group—Hui Chinese. The total population of Hui Chinese has reached approximate 9.8 million, and they use Mandarin mostly in their daily lives. However, Hui Chinese still use Arabic during some religious activities and communications owing to their belief in Islam. Globalization has brought about drastic cultural change within Chinese society, which has caused strong cultural shock among Tibetan and Hui ethnic minorities. In response, the phenomenon of cultural anxiety among these ethnic minorities has become increasingly pervasive [7]. For instance, films about such ethnic minorities’ cultures have themes involving various challenges, transformations, and fissions [23]. The stronger the cultural anxiety these minorities perceive, the more they will want to stick to their own ethnic identity. We thus selected samples of Tibetan and Hui people to test the hypotheses in the present study.

## Methods

### Ethics Statement

The study was reviewed and approved by the Committee of Protection of Subjects at Beijing Normal University. All participants provided written informed consent before the study and were fully debriefed at the end of the research according to the established guidelines of the committee.

### Participants

Sample 1 included 102 Tibetan college students from a Tibetan language and culture class at Minzu University of China and 124 Tibetan college students from ethnic minority classes at Beijing Normal University. All responses were valid. Among the participants, 45.1% were male and 54.9% were female, and ages ranged from 18 to 23 years old (*M* = 21.2, *SD* = 2.02). In terms of region of origin, 46.3% were from Tibetan-inhabited areas, and 53.7% were from areas where Tibetan and Han people live together. Subjects who communicated with their parents in Tibetan at home constituted 94.5% of the sample; 98% of them had parents belonging to the same nationality. Most subjects had lived in Han culture for 3 to 4 years. Participants had learned Chinese for an average of 14.5 years prior to the study.

Sample 2 included 102 Chinese Hui undergraduates randomly selected from ethnic minority classes at the Northwest Normal University. Participants were between 18 and 23 years of age (*M* = 22.1, *SD* = 1.98), and 76.5% were female. Of the 102 participants, 24.5% were from Hui-inhabited areas, and the rest came from areas where Hui and Han live together. Most participants (96.5%) had parents of the same nationality.

### Measures and Procedure

A set of three questionnaires was administered to all participants. The questionnaires were scored on a 7-point Likert scale with Tibetan participants and a 5-point Likert scale with Hui participants ranging from "totally disagree" to "totally agree". All questionnaires rigorously translated from English to Chinese and back-translated to English by psychologists. Students anonymously completed the questionnaires, which were presented in Chinese, during class. All participants read an informed consent document and received 5 Yuan RMB when they finished all of the questionnaires. Statistical analyses were undertaken by using SPSS 13.0 software.

1. Cultural anxiety questionnaire. This questionnaire was created by researchers for this study’s specific purpose based on the concept of cultural anxiety defined by Grillo [6] as well as related concepts in intercultural communication. The questionnaire included five items, namely, “I’m worried about the survival and development of Tibetan culture”, “Sometimes I have a strong sense of crisis about Tibetan culture”, “I'm concerned that one day Tibetan culture would lose its uniqueness”, “I'm worried about inheritance and protection of Tibetan culture”, and “It would be very unfortunate if Tibetan culture will be changed a lot due to external forces”. High scores indicated strong cultural anxiety. To test the reliability and validity of the self-composed questionnaire, we did a pre-test with 121 Tibetan students who were randomly selected from a minority class at a university. It was found that the internal consistency coefficient of the cultural anxiety questionnaire was 0.88. Confirmatory factor analysis showed that the single factor model had a good fit (*χ* ^*2*^ */ df* = 3. 85, *NFI* = 0.95, *CFI* = 0.97, *IFI* = 0.97, *GFI* = 0.96, *RMSEA* = 0.07). All factor loadings were between 0.60 and 0.86, which is psychometrically acceptable. In the formal study, the questionnaire’s α_Tibetan_ = 0.92 and α_Hui_ = 0.71.
2. Endorsement of a Multicultural Ideology Scale. This questionnaire [10] has 10 items. Five items are worded positively, such as, “Compared to having only one single ethnic culture, a society that consists of different ethnic groups would better handle continuous social issues”. The other five items are worded negatively and reverse scored, for example, “Chinese society would be better off if all people try to obscure their respective cultural background”. Higher scores indicate more support for a multicultural ideology, while lower scores indicate more support for an assimilation ideology. We have modified the scale to fit into the context of Chinese culture. A pre-test was also performed with 121 ethnic minority students randomly selected from a minority class at a university. The results showed that the internal consistency coefficient of the Endorsement of a Multicultural Ideology Scale was 0.68. Confirmatory factor analysis showed that the single factor model fit well (*χ* ^*2*^ */ df* = 3. 98, *NFI* = 0.90, *CFI* = 0.89, *IFI* = 0.89, *GFI* = 0.92, *RMSEA* = 0.08). All indicators reached the acceptable statistical level. In this formal study, the questionnaire’s α_Tibetan_ = 0.76 and α_Hui_ = 0.69.
3. Ethnic essentialism questionnaire. We used The Lay Theory of Ethnic Group Scale composed by No et al. [18]. The scale is used to measure whether people think there are essentially different ethnic categories. It has eight items, four items to measure ethnic essentialism such as, “How a person is like (e.g., his or her abilities, traits) is deeply ingrained in his or her race. It cannot be changed much”, and four items to measure social constructionism such as, “Racial categories are completely constructed based economic, political, and social reasons. If the socio-political situation changes, the racial categories will change as well”. Essentialism and social constructionism are two poles of the same dimension [18]; thus, we can compute the final score of ethnic essentialism by reversing the scores of social constructionism and integrating those scores with the first four items to synthesize into a single ethnic essentialism score. We also tested the reliability and validity of the questionnaire based on the pre-test with 121 Tibetan students who were randomly selected from a minority class at a university. The internal consistency coefficient of this scale was 0.76. Confirmatory factor analysis showed that the model had good fit (*χ* ^*2*^ */ df* = 4.36, *NFI* = 0.91, *CFI* = 0.89, *IFI* = 0.89, *GFI* = 0.90, *RMSEA* = 0.06). In the formal study, the questionnaire’s α_Tibetan_ = 0.81 and α_Hui_ = 0.76.

## Results

### 1. Preliminary analyses

The correlations between cultural anxiety, endorsement of a multicultural ideology and ethnic essentialism, and their means and standard deviations, with Sample 1 (Tibetan participants) and Sample 2 (Hui participants) are shown in Tables 1 and 2.

**Table 1:** Correlations between all Variables with Sample 1.

	^1^cultural anxiety	^2^the endorsement of a multicultural ideology	^3^ethnic essentialism
*M*	5.89	5.47	5.50
*SD*	1.22	0.91	0.95
2	0.59*		
3	0.43***	0.59***	

*p < .05;

**p < .01;

*** *p* < .001.

M = Mean; SD = Standard Deviation.

**Table 2:** Correlations between all Variables with Sample 2.

	^1^cultural anxiety	^2^endorsement of a multicultural ideology	^3^ ethnic essentialism
	3.47	3.95	3.98
*SD*	1.01	0.42	0.73
2	0.27**		
3	0.31***	0.39***	

*p < .05;

**p < .01;

*** *p* < .001.

M = Mean; SD = Standard Deviation.

The Pearson correlation analysis indicated that cultural anxiety, the endorsement of a multicultural ideology and ethnic essentialism were all positively correlated with each other (see Tables 1 and 2).

Furthermore, hierarchical regression was used to test the extent to which cultural anxiety and endorsement of a multicultural ideology could predict ethnic essentialism. To rule out the influence of school type, gender, and the region of origin, these factors were dummy coded and entered in the first step of the analysis. In the second step, cultural anxiety and endorsement of a multicultural ideology were included in the analysis after being centered, with ethnic essentialism serving as the dependent variable in the regression model. The results are shown in Tables 3 and 4.

**Table 3:** Hierarchical regression with Sample 1.

Predictive variables	Step 1			Step 2		
	*β*	*TOL*	*VIF*	*β*	*TOL*	*VIF*
gender	.12	.84	1.19	-.02	.80	1.26
region of origin	-.07	.80	1.25	-.07	.75	1.34
school type	.01	.94	1.06	.05	.94	1.06
cultural anxiety				.15*	.68	1.63
the endorsement of a multicultural ideology				.50***	.67	1.66
*R* ^*2*^	2.7%			36.2%		
*ΔR* ^*2*^	2.7%			33.5%		
*F*	2.03			24.98***		
*ΔF*	2.03			57.84***		

*p < .05;

**p < .01;

*** p < .001.

β = Regression Coefficient; TOL = Tolerance; VIF = Variance Inflation Factor; R^2^ = Coefficient of Determination; ΔR^2^ = R^2^ Change; F = F Value;ΔF = F Change.

**Table 4:** Hierarchical regression with Sample 2.

Predictive variables	Step 1			Step 2		
	*β*	*TOL*	*VIF*	*β*	*TOL*	*VIF*
gender	-.02	.97	1.03	.10	.81	1.23	
region of origin	-.05	.97	1.03	-.005	.97	1.03
cultural anxiety				.27**	.78	1.28	
endorsement ofa multicultural ideology				.32***	.92	1.09
*R* ^*2*^	0.3%			20.9%		
*ΔR* ^*2*^	0.3%			20.6%		
*F*	0.13			6.37***		
*ΔF*	0.13			12.59***		

*p < .05;

**p < .01;

*** p < .001.

β = Regression Coefficient; TOL = Tolerance; VIF = Variance Inflation Factor; R^2^ = Coefficient of Determination; ΔR^2^ = R^2^ Change; F = F Value;ΔF = F Change.

Based on the results of collinearity diagnostics, neither of the two predictors had a tolerance value smaller than 0.10 or a variance inflation factor (VIF) statistic greater than 10.0. These results indicated that there was not a significant multi-collinearity problem among the predictors in the model. Regression analysis revealed that after controlling for school type, gender, and region of origin, the regression model accounted for significantly more variance in the dependent variable (*F*(5, 220) = 24.98, *p* < .001; (*F*(4, 97) = 6.37, *p* < .001). As shown in Tables 3 and 4, cultural anxiety and endorsement of a multicultural ideology both positively predicted ethnic essentialism, and the total variance explained by the two predictors was 33.5% and 20.6%.

### 2. Mediation Analyses

To test the mediating effect of the endorsement of a multicultural ideology on the relationship between cultural anxiety and ethnic essentialism, regression analysis was conducted. We first tested the associations between ethnic essentialism, the endorsement of a multicultural ideology, and cultural anxiety. The results suggested that cultural anxiety positively predicted ethnic essentialism (*β*_Sample Tibetan_ = .44, *SE* = 0.05, *t*(226) = 7.22, *p* < .001; *β*_Sample Hui_ = .37, *SE* = 0.07, *t*(102) = 3.51, *p* < .001) and the endorsement of a multicultural ideology (*β*_Sample Tibetan_ = .59, *SE* = 0.04, *t*(226) = 10.95, *p* < .001; *β*_Sample Hui_ = .30, *SE* = 0.06, *t*(102) = 2.83, *p* < .01). Testing our meditation model, when cultural anxiety and the endorsement of a multicultural ideology were entered into the regression model predicting ethnic essentialism, the prediction coefficient of cultural anxiety declined greatly but was still significant (*β*_Sample Tibetan_ = .13, *SE* = 0.05, *t*(226) = 2.02, *p* < .05; *β*_Sample Hui_ = .27, *SE* = 0.07, *t*(102) = 2.62, *p* = .01), and the endorsement of a multicultural ideology was a significant predictor (*β*_Sample Tibetan_ = .51, *SE* = 0.07, *t* (226) = 7.65, *p* < .001; *β*_Sample Hui_ = .32, *SE* = 0.12, *t*(102) = 3.40, *p* < .001). Using the bootstrapping method (with 5,000 iterations) recommended by Preacher and Hayes [24], we tested the significance of the mediating effect. The 95% confidence interval for the indirect effect of the endorsement of a multicultural ideology did not include zero (range of Sample Tibetan: 0.18–0.38; range of Sample Hui: 0.008–0.19), suggesting that cultural anxiety not only influenced ethnic essentialism directly but also indirectly influenced it through the endorsement of a multicultural ideology.

## Discussion

Our results showed that cultural anxiety, the endorsement of a multicultural ideology, and ethnic essentialism were all positively correlated with each other from the perspective of the minority groups. The endorsement of a multicultural ideology partially mediated the relationship between cultural anxiety and the endorsement of a multicultural ideology across two different ethnic groups (Tibetan and Hui). Therefore, all of our hypotheses were verified. Taken together, our results indicated that when cultural anxiety was high, the endorsement of a multicultural ideology and ethnic essentialism were stronger. In addition, the endorsement of a multicultural ideology was the most direct and adjacent factor that reinforced ethnic essentialism.

In an environment that is in general shared with Han culture, Tibetan and Hui people feel an acculturation of stress from Han culture, with cultural anxiety occurring naturally. The best way to maintain a cultural identity is to support a multicultural ideology in the same political system [21, 22]. Supporting a multicultural ideology could highlight the difference between minority cultures and Han culture. Meanwhile, increasing the psychological boundary between the minority group and Han group would also clarify the former as in fact a group. Therefore, when cultural anxiety increases, there is greater support for the endorsement of a multicultural ideology, which coincides with greater ethnic essentialism; the endorsement of a multicultural ideology is the intermediary variable between the cultural anxiety and ethnic essentialism.

Verkuyten [2] conducted a study using discourse analyses and found that minority groups tended to essentialize their identities when discussing assimilation and identity denial. If minority groups’ identities are ignored (e.g., perceiving the majority group members’ views of assimilation), they will exploit essentialism to affirm or highlight their identities. This suggests that minority groups’ ethnic essentialism will be strengthened by anxiety about their culture or their identity. Research also suggests that stronger cultural anxiety may protect and support a multicultural ideology among minorities. When ethnic minorities experience cultural anxiety, they feel a need to keep their culture from disappearing or being contaminated, and they enhance awareness about adopting a multicultural ideology [11, 21]. Our research also revealed that cultural anxiety not only influenced ethnic essentialism directly but also influenced it indirectly through the endorsement of a multicultural ideology. Previous studies have shown that maintaining a multicultural ideology is the basis of essentialist beliefs. Endorsing a multicultural ideology can serve to mark unique ethnic identities for minorities [5, 10]. Thus, endorsing a multicultural ideology was positively correlated with essentialist beliefs. From this perspective, ethnic essentialism also has positive implications because it benefits the preservation and inheritance of ethnic culture and ethnic identity. In a society where the Han culture is prevalent and ethnic minorities’ cultures are shared only by minority groups, the more cultural anxiety ethnic minorities feel, the more they will protect a multicultural ideology and the stronger their ethnic essentialism will become. Our results indicate that ethnic essentialism becomes stronger with increases in the endorsement of a multicultural ideology. The results are consistent with Verkuyten’s findings [2, 22], which reveals that ethnic essentialism is indeed an issue of ideology to a large extent.

The findings have important theoretical implications. Many past studies have explored group essentialism’s influence on society, using essentialism as an independent variable [3, 4, 5]. In contrast, we treated ethnic essentialism as a dependent variable and examined the factors that influenced it. Moreover, past research has demonstrated that minority members’ essentialism has positive implications for the expression of identity. For instance, Verkuyten [2] found that in terms of race, minorities’ essentialism will be enhanced under conditions of assimilation. Another study conducted by Morton and Postmes [25] reported that homosexuals’ essentialism of sexual orientation was enhanced when they perceived their identities to be ignored or denied. However, in our research, cultural anxiety was defined broadly, such that when ethnic minorities perceive that their culture is ignored, denied, marginalized, changed, or threatened, they will feel cultural anxiety. This approach enlarges the generalization of previous findings. Another contribution of this study is that we conducted the research in Chinese culture, and we tested whether the cultural anxiety of ethnic minorities not only influenced ethnic essentialism directly but also influenced it indirectly through the endorsement of a multicultural ideology. Our findings also expand previous research by identifying mediators to explain existing theoretical frameworks.

The findings have important practical implications. It is worth noting that high ethnic essentialism will cause negative intergroup relationships between minority groups and majority groups [15, 17]. This study has found that cultural anxiety, along with the endorsement of a multicultural ideology, is an influencing factor of ethnic essentialism. Therefore, to promote inter-ethnic relationships in China, we should take measures to enhance the protection and inheritance of minority cultures and reduce sources of cultural anxiety, such as ignoring, denying, marginalizing, changing, or threatening minority cultures, in order to protect the multicultural environment.

Our research also has limitations. First, the language of the research material was Mandarin, as opposed to minority languages, which may have influenced the results. Research has found that language is one of the principal elements that impacts cultural identity [26]. Second, this research is a correlational study, so it cannot explain the causal relationships among the three factors. Future studies may explore the causal relationships among these three factors by adopting an experimental method or longitude study. Third, this study has explored the mediation mechanism between cultural anxiety and ethnic essentialism from the perspective of minority groups. Future study may further explore the issue from the perspective of a majority group. At last, we have not explored other factors that may influence ethnic essentialism, such as the need for cognitive closure, the fear of illness and death, and social dominance orientation [5]. We hope to explore these social psychological mechanisms in future research.

## Supporting Information

S1 Dataset The data for study with Sample 1. (SAV)

S2 Dataset The data for study with Sample 2. (SAV)
